# Spinal teratoma in an adolescent: a rare case report with intradural presentation and review of management strategies

**DOI:** 10.1097/MS9.0000000000003561

**Published:** 2025-07-09

**Authors:** Sudarsan Agarwal, Reshma Agarwal, Sameer Kumar Majety, Ananta Kiran Kumar Veldurthi, Santhoshi Devi Koppala, Gopichand Muppana

**Affiliations:** a Department of Neurosurgery, Sri Venkateswara Medical College, Tirupati, India; b School of Medicine, China Medical University, Shenyang, P.R. China; c School of Medicine, Xiamen University, Xiamen, P.R. China; d Department of Radiology, Sri Venkateswara Medical College, Tirupati, India; e National Pirigov Memorial Medical University, Vinnytsia, Ukraine

**Keywords:** aseptic meningitis, intradural tumor, monodermal teratoma, spinal teratoma, surgical excision

## Abstract

**Introduction:**

Spinal teratomas are rare tumors of pluripotent germ cells, accounting for <0.5% of all spinal cord tumors and 2% of all teratomas. While they commonly occur in gonads, extragonadal spinal presentation is uncommon. They are often associated with spinal dysraphism and present variably depending on tumor location and neural compression. MRI aids in diagnosis, but histopathological examination remains the gold standard. Early detection is vital to avoid irreversible neurological damage.

**Case presentation:**

A 16-year-old Indian male presented with progressive lower back pain for one year, followed by involuntary micturition and bilateral temporal headaches for six months. Neurological examination was normal, but persistent urinary symptoms warranted imaging. MRI revealed an intradural lesion at D11–L1, consistent with a spinal teratoma. The patient underwent surgical excision, and histopathology confirmed a monodermal teratoma. At three-month follow-up, the patient’s symptoms had completely resolved.

**Discussion:**

Spinal teratomas may be classified as mature, immature, or malignant. Mature teratomas are most common in adults. Theories regarding their origin include misplaced primordial germ cells and dysembryogenic malformations. Clinical presentation varies from pain to autonomic dysfunction, demanding high clinical suspicion and prompt imaging. Surgical excision remains the mainstay of treatment. Subtotal resection is considered when tumors adhere to critical neural structures. Although rare, recurrence, malignant transformation, and aseptic meningitis have been reported, emphasizing the need for long-term follow-up.

**Conclusion:**

This case underscores the importance of early neuroimaging in patients with atypical spinal symptoms. Surgical resection is definitive, while histopathology confirms the diagnosis. Regular follow-up remains essential.

## Introduction

Spinal Teratomas are rare tumors that arise from pluripotent germ cells and contain tissues derived from one or more of three germ layers: ectoderm, mesoderm, endoderm^[^1^]^. While teratomas are more commonly found in gonads, extragonadal occurrence, especially in the spine, is extremely rare. Approximately <0.5% of all spinal cord tumors and 2% of overall teratomas constitute for spinal teratomas^[^2^]^. Spinal teratomas are incredibly rare, as evidenced by several studies. In 1964, Slooth *et al* examined 1322 cases of spinal tumors and found only two instances of spinal teratomas. Likewise, over a 15-year period, Al-Sarraj *et al* reviewed 25 000 neurosurgical biopsies but identified just seven cases, emphasizing how uncommon these tumors truly are^[^3^]^.

**HIGHLIGHTS**

- Early MRI helped identify a rare spinal teratoma in an adolescent male patient.
- Histopathology confirmed a monodermal teratoma after complete surgical excision.
- Spinal teratomas may present with pain, urinary symptoms, or autonomic features.
- Subtotal resection may be preferred if tumor is attached to vital neural structures.
- Long-term follow-up is key due to rare but possible recurrence or complications.

Spinal teratomas most commonly occur in young children and are especially associated with vertebral and spinal dysraphism^[^2^]^. Based on the location of the mass in the spinal cord, they may present with a variety of symptoms. Considering a spinal cord tumor as a differential diagnosis in cases of backache or radiating pain caused by cord or nerve compression, with or without neurological deficits, is crucial^[^4^]^. Although imaging studies like CT and MRI play a vital role, histopathological examination following surgical resection serves as the gold standard for the diagnosis of this disease^[^2^]^. Demonstrating the presence of pluripotent cells from one (monodermal) or more germ layers confirms the diagnosis of a spinal teratoma^[^1^]^.

This 16-year-old patient’s presentation with nonspecific symptoms of lower back pain, involuntary micturition, and headache highlights the atypical manifestations of spinal teratoma. Recognizing such signs play a crucial role in early diagnosis and intervention, preventing irreversible neurological damage. Studying these atypical cases can help refine diagnostic criteria, improve imaging-based detection, and enhance timely surgical management, ultimately leading to better patient outcomes.

## Case presentation

A 16-year-old Indian boy presented with a one-year history of persistent lower back pain that had been gradually increasing in severity. Over the past six months, he had developed involuntary micturition, followed by the onset of bilateral temporal headaches in the last month. Additionally, the patient reported generalized fatigue. He had no history of trauma, prior neurological conditions, or systemic illness.

Even though examination revealed no neurological deficits, his persistent urinary symptoms and worsening pain warranted further investigation. Laboratory workup showed a hemoglobin level of 13.2 g/dL, an RBC count of 2.64 million/mm^3^, and a WBC count of 8500 cells/mm^3^, with a differential count of 44% neutrophils, 49% lymphocytes, 5% eosinophils, and 2% monocytes. Platelet levels were elevated at 4.10 lakhs/mm^3^. Liver and renal function tests, as well as serum electrolytes, were within normal ranges.

**Figure 1.**
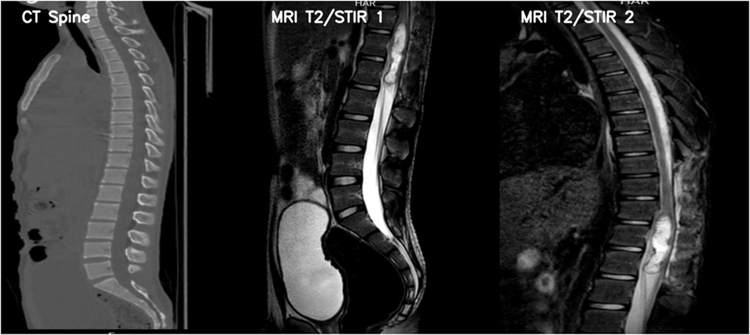
Spinal imaging showing an intradural lesion. (Top) CT scan reveals an increased spinal canal diameter from D11 to L1. (Middle & bottom) MRI T2/STIR sequences demonstrate a well-defined hyperintense lesion, suggestive of a cystic or fatty component, characteristic of an intraspinal teratoma.

Considering the chronic and persistent nature of the symptoms, neuroimaging was pursued. A CT scan (Fig. 1) of the lumbar spine demonstrated an increased spinal canal diameter from D11 to L1. MRI of the lumbar spine (Fig. 1) revealed a well-defined, intradural lesion extending from D11 to L1, measuring approximately 6.5 × 8 × 1.5 cm. On T2-weighted, T1-weighted, and STIR sequences, it appeared as heterogeneous hyperintensity, with evidence of restricted diffusion on DWI. These findings were indicative of an intradural extramedullary (IDEM) spinal cord tumor. Based on the imaging findings and the patient’s symptoms, the differential diagnoses included spinal teratomas, dermoid and epidermoid cysts, schwannomas, meningiomas, neurofibromas, metastatic lesions, lipomas, and arachnoid cysts.

Due to the potential risk of neurological deterioration, after a thorough preoperative evaluation, surgical excision of the mass was performed under general anesthesia. The patient was positioned prone, and a laminectomy from D10 to L2 was performed. The dura was opened using a standard technique, and durotomy exposed the conus–cauda equina junction with an intradural mass containing hair, sebaceous material, and tooth particles. Subtotal excision of the tumor was performed, as it was found to be intermingled with neural elements. The tumor was carefully dissected from surrounding nerve roots, and piecemeal removal was achieved. Although intraoperative neuromonitoring was not available in this limited-resource setting, potential damage to the nerve roots was considered a key complication. Postoperative assessment of nerve reflexes was conducted prior to discharge to ensure no functional neurological deficit. Additionally, the patient was monitored for any incipient bowel/bladder complications. Methylprednisolone (1 g) was administered to prevent chemical meningitis, and the dura was closed in a watertight fashion. The mass was excised without any intraoperative complications.

**Figure 2.**
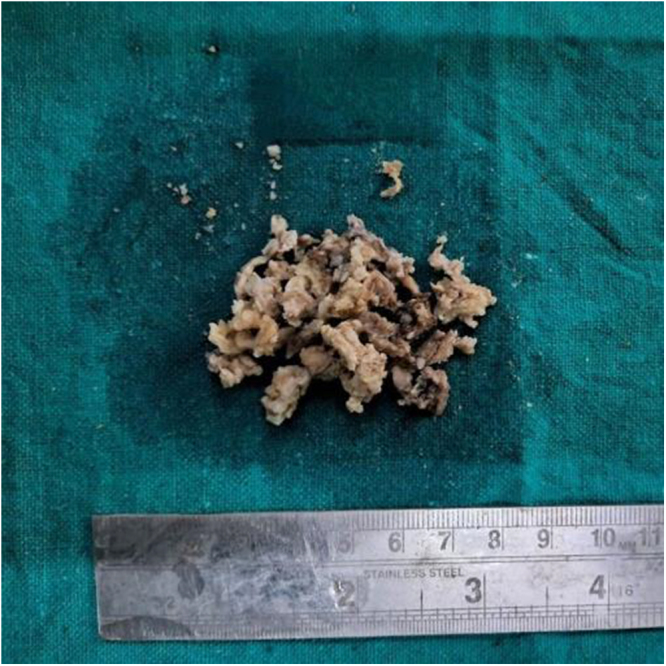
Gross specimen measuring 4 × 3 × 2 cm, showing fragmented, irregularly shaped, tan-white and brown material, consistent with keratinous debris, suggestive of a mature teratoma.

**Figure 3.**
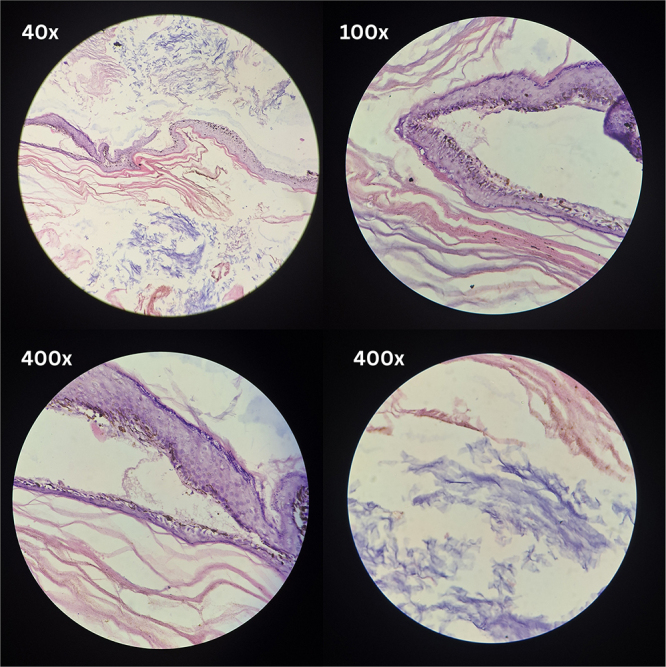
Histological sections of a mature teratoma at different magnifications. (Top left) 40×: low-power view showing heterogeneous tissue composition. (Top right) 100×: stratified squamous epithelium with underlying stroma. (Bottom left) 400×: keratinized squamous epithelium. (Bottom right) 400×: keratin debris.

Histopathological examination of the excised specimen (Fig. 2), consisting of multiple friable, grey-brown soft tissue fragments measuring 4 × 3 × 2 cm, confirmed the diagnosis. Microscopic sections (Fig. 3) demonstrated a cyst lined by keratinized stratified squamous epithelium, with luminal lamellated keratin material, consistent with a dermoid cyst or monodermal teratoma.

At a follow-up visit three months after the surgery, the patient appeared to be recovering as expected without any signs of complications. His symptoms of involuntary micturition, persistent lower back pain, and temporal headaches had subsided.

This report represents a unique case of a monodermal teratoma at the D11–L1 spinal level. The successful surgical excision of a spinal teratoma, which is very rare in clinical practice, highlights the importance of early imaging in patients presenting with chronic spinal pain and urinary dysfunction, ensuring timely intervention and optimal neurological outcomes.

## Case discussion

Teratoma is defined as a cystic or solid tumor with pluripotent germ cells from one or more germ layers^[^5^]^. Teratomas most commonly occur in the coccyx (44.5%) and ovaries (42.1%), followed by the head and neck^[^1^]^. Spinal teratomas are very rare and accounting for only 2% of all teratomas and <0.5% of intraspinal tumors^[^2,3^]^. Based on the degree of differentiation and types of tissue present, teratomas can be classified as mature, immature, or malignant^[^4^]^. Spinal teratomas in the adult population are rare, predominantly of the mature subtype, and tend to occur at the level of the conus medullaris. In contrast, pediatric cases are more likely to involve other subtypes and occur at the sacrococcygeal level^[^1^]^.

Mature teratomas contain well-differentiated tissues such as bone, teeth, hair, cartilage, glands, muscle, and neural elements. Immature teratomas contain more primitive, embryonic tissue. Malignant teratoma**s** consist of both mature and immature subtypes, along with fragments from germ cell tumors such as yolk sac tumor and choriocarcinoma, which are associated with high levels of serum α-fetoprotein^[^4,5^]^. Immature teratomas tend to be aggressive and have a greater tendency to recur, while malignant teratomas are most commonly associated with a poor prognosis^[^4^]^.

The gender distribution of spinal teratomas remains inconclusive due to the rarity of reported cases. A study conducted by Harms *et al*, inclusive of 170 cases, revealed a female-to-male ratio of 1.3:1, with sacrococcygeal teratomas being the most predominant type^[^6^]^. However, the generalizability of this study is questionable, as it included a variable proportion of mature, immature, and malignant teratomas. Another study indicated even higher female predominance, with a ratio of 5.5:1 among Black patients, with sacrococcygeal teratomas being the most common type^[^7^]^. However, these findings do not correlate with our case, as our patient is an adolescent male.

Tumors of the spinal cord are classified as extradural and intradural, which can be further classified into extramedullary or intramedullary. Intradural extramedullary (IDEM) tumors are the most common spinal tumors (**55%**). Although there are a wide variety of IDEM tumors, IDEM teratomas are rare in adults^[^4^]^ compared to intradural intramedullary (IDIM) teratomas^[^8^]^.

To date, no definitive genetic basis has been established for spinal teratomas, and available data are limited to experimental studies and isolated case reports. While mature spinal teratomas are primarily thought to arise from the developmental misplacement of pluripotent germ cells, emerging evidence suggests a possible genetic contribution, especially in syndromic or familial presentations. Chromosomal abnormalities identified in sacrococcygeal teratomas include distal 10q trisomy with partial 17p monosomy and mosaic trisomy of chromosome 1q, suggesting that genomic instability may play a role in tumor development^[^9^]^. In Currarino syndrome, which often presents with presacral teratomas and sacral dysgenesis, mutations in the HLXB9 gene located on chromosome 7q have been described, indicating a potential genetic link in select cases^[^9^]^. Additionally, a study using whole-exome sequencing of mature ovarian teratomas identified recurrent mutations in genes such as FLG, MUC17, MUC5B, and GOLGA6L2, with novel mutations in DUSP5 and PHLDA1 potentially contributing to unique tumor characteristics^[^10^]^. Our case did not undergo genetic testing, and therefore no molecular correlation could be assessed.

We describe a case of intradural extramedullary (IDEM) spinal cord tumor at the level of D11–L1 in a 16-year-old male. Although teratoma is diagnosed based on the presence of pluripotent germ cells from all three germ layers, occasionally there can be dominance of cells from one or more layers. As a result, the diagnosis cannot be ruled out if the tissue specimen does not contain cells from all three germ layers^[^1^]^. The presence of cysts lined by keratinized stratified squamous epithelium with luminal lamellated keratin material in this case is consistent with the histopathological diagnosis of a teratoma. This spinal tumor, with cells derived from a single germ layer, can be considered a monodermal spinal teratoma, a rare entity^[^11^]^.

Understanding the pathogenesis of spinal teratoma has become crucial considering its rarity, limited research, and atypical presentation. The two most popular theories for the origin of spinal teratoma are the dysembryogenic theory and misplaced primordial germ cell theory. According to Wan *et al*, dysraphic malformations strongly support the dysembryogenic theory, where spinal teratomas form from disordered differentiation of pluripotent cells of the primitive streak or caudal cell mass due to impaired gene functions and cellular inductive interactions. Conversely, the absence of dysraphism in many adult intradural teratomas supports the misplaced germ cell theory, suggesting that spinal teratomas arise from pluripotent primordial germ cells of the neural tube that are misplaced into the dorsal midline during their normal migration from the primitive yolk sac to the gonadal ridges. Based on their research on 18 cases, Wan *et al* proposed that the theory of Makary and colleagues^[^12^]^ is the most acceptable at present, as it considers the cause-and-effect relationship between spinal cord malformation and migration of primordial germ cells. This theory proposes that due to the impaired genetic and cellular inductive interactions during embryogenesis, the pluripotent cells may get trapped in an abnormal environment and undergo disorderly differentiation, resulting in the formation of a teratoma^[^8^]^.

As far as the clinical presentation of spinal teratomas (STs) is considered, it is highly variable in adults. They can range from chronic pain to neurological deficits to autonomic irregularities. The most common location for STs to occur is in the **t**horacolumbar region, especially the conus ^[^13^]^. This was observed in our patient, who presented with chronic pain and autonomic symptoms like involuntary micturition. External anomalies may also suggest the location of a teratoma, as they are sometimes associated with defects such as dimples, dermal sinuses, hair tuft**s**, and overt vertebral anomalies at the site^[^8^]^.

Even though the symptom profile in adults can be variable, one could say that it is more difficult to diagnose a spinal teratoma in an infant. Rainey *et al* reported one such case where an infant with a spinal teratoma presented with progressive feeding intolerance, which could have been caused by many conditions^[^5^]^. In the aforementioned case, feeding intolerance was accompanied by abnormal reflexes, which prompted a neurological diagnosis. CT brain and MRI brain did not reveal any obvious pathology, after which lumbar spine MRI was performed due to an abnormal lumbar puncture. The patient was then promptly treated with surgical resection and adjuvant chemotherapy. This case serves as an example where the clinician was able to help the patient despite atypical symptoms. Weakness of the lower limbs, sensory, and reflex abnormalities are the most common symptoms, which may occur in a progressive or intermittent-progressive fashion, according to a study of 31 patients^[^14^]^. There can also be cases where the patient suffers primarily from autonomic symptoms with minimal to no motor involvement^[^3^]^.

All the examples stated above elucidate the importance of diagnostics in a case of **s**pinal teratoma due to its nonspecific and variable symptoms. The mainstay in diagnosing an ST is neuroimaging with computed tomography (CT) or magnetic resonance imaging (MRI)^[^13^]^. Even though CT is a sensitive test, it is largely restricted to teratomas with heterogeneous components like fat, calcifications, and cysts^[^14^]^. MRI of the spine is the preferred imaging modality, where both heterogeneous and homogeneous aspects of the mass can be evaluated efficiently. Spinal teratomas usually appear as a combination of solid and cystic components on MRI, often containing fat and calcifications. Mature teratomas generally lack significant contrast enhancement, but some of them might exhibit peripheral rim enhancement or focal nodular enhancement as an atypical feature^[^13^]^. Our patient’s MRI revealed a well-defined hyperintense lesion at the D11–L1 level, correlating with his symptoms and prompting surgical excision.

Although imaging and symptoms can help guide preoperative planning, the definitive diagnosis of spinal teratoma is almost always established through histopathological examination (HPE) of the excised specimen^[^13^]^. Radiologically, the differential diagnoses for intradural-extramedullary (IDEM) masses include common tumors such as schwannomas, meningiomas, neurofibromas, and metastatic lesions, as well as rarer entities like dermoid and epidermoid cysts, lipomas, and arachnoid cysts^[^15^]^. IDEM lesions are situated within the dura but outside the spinal cord, often displacing the cord and expanding the thecal sac. On MRI, schwannomas typically appear isointense on T1 and hyperintense on T2, with homogeneous or heterogeneous post-contrast enhancement and, occasionally, a “target sign.” Meningiomas often demonstrate uniform enhancement and isointensity on both T1- and T2-weighted images. Epidermoid cysts closely mimic cerebrospinal fluid across sequences but are distinguished by their marked diffusion restriction on DWI. In contrast, lipomas are hyperintense on T1, suppressed on fat-saturated sequences, and lack complex internal architecture. In our case, the lesion demonstrated heterogeneous signal intensity on both T1- and T2-weighted images, with clear fat components and partial calcifications, which raised the suspicion of a teratomatous lesion. Intraoperatively, the presence of hair, sebaceous material, and tooth-like structures further supported this impression. Histopathological examination confirmed the diagnosis as a mature monodermal teratoma, allowing for definitive classification and guiding further management^[^16^]^.

While histopathology confirmed a mature monodermal teratoma, tumor markers such as serum alpha-fetoprotein (AFP) and beta-human chorionic gonadotropin (β-HCG) were not evaluated in this case due to limitations in diagnostic resources. β-HCG is particularly relevant in identifying choriocarcinomatous components, and AFP is typically elevated in yolk sac tumors^[^17^]^. Although the lesion was radiologically and histologically consistent with a mature teratoma, the absence of tumor marker data limits the ability to definitively exclude the presence of mixed or malignant germ cell elements.

A recent study conducted in 2024, which analyzed 38 previously published cases, stated that gross surgical excision of the tumor is the most employed treatment strategy^[^18^]^. Supporting this, another study of 27 patients over a 20-year period mentioned that surgical excision must be performed whenever possible^[^19^]^. In this study by Sharma *et al*, complete surgical excision was achieved in 15 patients and subtotal excision in 12 patients. Subtotal excision is generally performed in patients where the tumor cannot be easily accessed or when it is adherent to critical neurological structures. The difference in recurrence rates between total excision and subtotal excision was negligible. Contrary to general expectations, subtotal resection indicated better neurological recovery. This may be due to the preservation of key neurological structures and the slow-growing nature of the spinal teratoma^[^20^]^. In our case, subtotal excision was performed due to the tumor’s adherence to the surrounding neural elements, prioritizing the preservation of neurological function.

The role of adjuvant therapy for a mature spinal teratoma still remains questionable, as there are no large-scale studies indicating a better prognosis when chemotherapy or radiotherapy are added. In contrast, immature or malignant teratomas strictly warrant the prompt initiation of adjuvant chemotherapy and radiotherapy^[^21^]^. Adjuvant chemotherapy can also be considered if a mature teratoma occurs in conjunction with another spinal cord tumor, like a yolk sac tumor^[^22^]^.

The role of adjuvant therapy for a mature spinal teratoma still remains questionable, as there are no large-scale studies indicating a better prognosis when chemotherapy or radiotherapy are added. In contrast, immature or malignant teratomas strictly warrant the prompt initiation of adjuvant chemotherapy and radiotherapy^[^21^]^. Adjuvant chemotherapy can also be considered if a mature teratoma occurs in conjunction with another spinal cord tumor, like a yolk sac tumor^[^22^]^. Following surgical resection, early postoperative rehabilitation is strongly recommended to optimize neurological recovery and functional independence. Physical and occupational therapy programs tailored to the patient’s deficits have been shown to improve mobility, reduce pain, and enhance quality of life in spinal tumor cases^[^23^]^. Structured inpatient rehabilitation, including progressive mobilization and targeted exercises, also supports prevention of complications such as muscle atrophy and contractures^[^23^]^.

Recurrence following surgical resection of spinal teratomas is relatively uncommon but appears influenced by factors such as the extent of excision and intraoperative capsule integrity. Allsopp *et al* reported similar recurrence rates – 9–10% after total resection and 10–11% after subtotal resection^[^21^]^. Conversely, Wan *et al* documented a 50% recurrence rate among patients who had subtotal resections, while no recurrences occurred in those who underwent complete excision^[^8^]^. Some studies also suggest that recurrence may be more common in cases where the tumor capsule is ruptured intraoperatively, potentially leaving behind microscopic residual tissue^[^24^]^. Postoperative radiological surveillance, particularly within the first five years, is therefore critical to detect early signs of recurrence. MRI is preferred for follow-up imaging due to its superior ability to delineate spinal tissue and detect small residual or recurrent lesions. Although the overall risk remains low, recurrence may present late and can mimic new neurological deficits or progressive symptoms, requiring renewed evaluation and possible intervention.

Although mature spinal teratomas are typically benign, documentation of malignant transformation exists, albeit infrequently. Estimates place the risk between 0.17% and 2%^[^25^]^, with squamous cell carcinoma being the most common malignant histology, followed by adenocarcinoma and sarcoma. Malignant transformation is more commonly reported in adult patients, with longer-standing lesions posing greater risk^[^26^]^. Age over 50 years and tumor size greater than 10 cm have been identified as particularly strong predictors of malignant transformation in extragonadal teratomas^[^27^]^. Late diagnosis or misinterpretation as benign lesions may delay intervention and increase the likelihood of malignant evolution. While our case did not exhibit malignant features on histology, awareness of this rare possibility remains critical. In confirmed cases of malignant transformation, the management paradigm shifts, with complete surgical excision followed by appropriate adjuvant chemotherapy and/or radiotherapy being recommended to reduce the risk of recurrence or metastatic spread.

An interesting and yet rare presentation of an intradural spinal teratoma can be recurrent aseptic meningitis. The intradural release of keratin, cholesterol, and lipoid materials might lead to the development of chemical meningitis^[^8^]^. More than one instance of such cases has been reported^[^28,29^]^. The case by Garg *et al*^[^29^]^ was initially thought to be bacterial meningitis, but paradoxical worsening secondary to cessation of corticosteroids prompted further investigation. Although a complete rupture of the teratoma, like the above case, might cause a severe and persistent presentation, minor leakages of material from teratomas could result in unexplained attacks of recurrent meningitis. These atypical presentations can challenge a doctor’s diagnostic skills at critical moments, making it essential for all healthcare professionals to be aware of them.

Given the potential for late recurrence even after gross total resection of spinal teratomas, we have planned a structured long-term follow-up strategy^[^21^]^. The patient will undergo MRI surveillance every six months for the first two years, followed by annual imaging for at least five years. Regular neurological examinations will be conducted to monitor for any signs of delayed recurrence, malignant transformation, or autonomic dysfunction, including bowel or bladder disturbances. As this is a rare entity, no standardized surveillance guidelines exist. Therefore, our follow-up protocol is adapted from existing case series and expert recommendations. In our case, only subtotal excision was achieved due to tumor adherence to critical neural elements, which carries a reported recurrence rate of 10–11%^[^21^]^. This further underscores the importance of diligent, long-term postoperative monitoring.

As a clinician, it is essential to recognize that even with appropriate surgical management, spinal teratomas carry a risk of recurrence and, in rare instances, malignant transformation. This reinforces the need for consistent postoperative surveillance and long-term clinical follow-up. Cases with atypical presentation or incomplete resection demand close multidisciplinary coordination between neurosurgery, oncology, and pathology teams to evaluate recurrence risk and determine the need for additional intervention. Early identification and timely response to subtle signs of progression are critical to optimizing neurological outcomes in these patients.

## Conclusion

Spinal teratomas are rare, particularly in adolescents, and often present with nonspecific neurological symptoms that delay diagnosis. Early consideration of such tumors in the differential diagnosis is crucial for timely intervention to avoid permanent neurological damage. Total or subtotal surgical resection remains the treatment of choice, with histopathology confirming the diagnosis. This case adds to the limited literature on spinal teratomas and reinforces the need for increased clinical awareness, prompt investigation of atypical symptoms, and structured follow-up. Given the risk of recurrence, especially in cases of subtotal excision, long-term surveillance with periodic MRI and neurological evaluation is essential to ensure early detection of delayed progression and to guide further management in this rare entity.

## Limitations

This case report has a few limitations. First, since spinal teratomas are extremely rare, a single case may not be broadly applicable to other patients. The limited number of reported cases also makes it difficult to establish definitive management guidelines. Second, while histopathology confirmed the diagnosis of a monodermal teratoma, we did not perform genetic or molecular analysis, which could have provided more insight into its origin and any possible congenital associations. Third, although the patient showed complete recovery at three months, long-term follow-up is necessary to assess the risk of recurrence or malignant transformation. Additionally, serum AFP and β-HCG levels were not measured, which might have offered further information on malignancy potential. Lastly, while surgical excision remains the preferred treatment, there is still uncertainty regarding the need for adjuvant therapy in cases with high-risk features, emphasizing the need for more studies on long-term outcomes.

## Data Availability

The data regarding the patient can be submitted by the corresponding author upon reasonable request.

## Research registration unique identifying number (UIN)

Not available for case report.
